# The Arf-GEF GBF1 undergoes multi-domain structural shifts to activate Arf at the Golgi

**DOI:** 10.3389/fcell.2023.1233272

**Published:** 2023-09-07

**Authors:** Justyna M. Meissner, Katarina Akhmetova, Tomasz Szul, Ekaterina G. Viktorova, Bingdong Sha, Jay M. Bhatt, Eunjoo J. Lee, Richard A. Kahn, George A. Belov, Igor Chesnokov, Elizabeth Sztul

**Affiliations:** ^1^ Department of Cell, Developmental and Integrative Biology, University of Alabama at Birmingham, Birmingham, AL, United States; ^2^ Department of Biochemistry and Molecular Genetics, University of Alabama at Birmingham, Birmingham, AL, United States; ^3^ Department of Veterinary Medicine, Virginia-Maryland Regional College of Veterinary Medicine, University of Maryland, College Park, MD, United States; ^4^ Department of Biochemistry, Emory University School of Medicine, Atlanta, GA, United States

**Keywords:** Sec 7 guanine nucleotide exchange factor, GBF1, Golgi, HDS3 domain, ARF

## Abstract

Golgi homeostasis require the activation of Arf GTPases by the guanine-nucleotide exchange factor requires GBF1, whose recruitment to the Golgi represents a rate limiting step in the process. GBF1 contains a conserved, catalytic, Sec7 domain (Sec7d) and five additional (DCB, HUS, HDS1-3) domains. Herein, we identify the HDS3 domain as essential for GBF1 membrane association in mammalian cells and document the critical role of HDS3 during the development of *Drosophila melanogaster*. We show that upon binding to Golgi membranes, GBF1 undergoes conformational changes in regions bracketing the catalytic Sec7d. We illuminate GBF1 interdomain arrangements by negative staining electron microscopy of full-length human GBF1 to show that GBF1 forms an anti-parallel dimer held together by the paired central DCB-HUS core, with two sets of HDS1-3 arms extending outward in opposite directions. The catalytic Sec7d protrudes from the central core as a largely independent domain, but is closely opposed to a previously unassigned α-helix from the HDS1 domain. Based on our data, we propose models of GBF1 engagement on the membrane to provide a paradigm for understanding GBF1-mediated Arf activation required for cellular and organismal function.

## Introduction

The Golgi complex plays a key role in the processing and post-translational modifications of newly synthetized proteins destined for secretion or in transit to other membranous organelles of the secretory and endo-lysosomal pathways (Farquhar and Palade, 1981; Pelham, 2001; Storrie, 2005; Glick and Nakano, 2009; Wilson et al., 2011; Barlowe and Miller, 2013). Golgi biogenesis and maintenance, as well as cargo protein transport from the ER to the Golgi are critically dependent on the activity of the ADP-ribosylation factor (Arf) family of small GTPases (Randazzo et al., 2000). Arfs mediate Golgi to ER recycling traffic, necessary for sustained forward membrane flow, by facilitating the recruitment of the COPI coating complexes that sort proteins into the recycling, or retrograde, pathway.

Arfs cycle between a cytosolic, inactive state when bound to GDP and a membrane-associated, active state when bound to GTP. Only the GTP-bound, active Arfs recruit the COPI coatomer to facilitate Golgi to ER retrograde traffic. Arfs have low intrinsic ability to displace GDP to allow GTP binding, and in cells, this reaction is promoted through an interaction with members of a family of guanine nucleotide exchange factors (GEFs) that catalyze GDP expulsion from the Arfs (Anders and Jurgens, 2008; Bui et al., 2009). The displacement reaction is mediated by a highly conserved ∼200 amino acid Sec7 domain (Sec7d) that is present in all Arf GEFs. This domain contains the catalytic “glutamic finger” residue that participates in the physical removal of the GDP from the Arf substrate (Beraud-Dufour et al., 1998; Renault et al., 2003). Arf activation at compartments of the ER-Golgi interface, such as ER exit sites (ERES) and ER-Golgi Intermediate Compartment (ERGIC), and within the Golgi is mediated by the Arf GEF GBF1 (Kawamoto et al., 2002; Alvarez et al., 2003; Garcia-Mata et al., 2003). GBF1 is ancient, as it is predicted to be present in the last eukaryotic common ancestor (LECA), and orthologs of GBF1 are expressed in all eukaryotic cells (Pipaliya et al., 2019). Most organisms have a single GBF1, but the yeast *S. cerevisiae* has two GBF1 orthologs named Gea1p and Gea2p. The requirement for GBF1 in mammalian cells is absolute, as inactivation of GBF1 through mutagenesis or by treating cells with Brefeldin A (BFA), a fungal metabolite inhibitor, leads to the collapse of the Golgi into the ER, inhibition in secretory traffic, induction of ER stress, and cell death (Claude et al., 1999; Kawamoto et al., 2002; Garcia-Mata et al., 2003; Zhao et al., 2006; Szul et al., 2007; Saenz et al., 2009). Similarly, the simultaneous deletion of *Gea1* and *Gea2* causes yeast cell death (Peyroche et al., 2001; Peyroche and Jackson, 2001; Spang et al., 2001).

**FIGURE 1:**
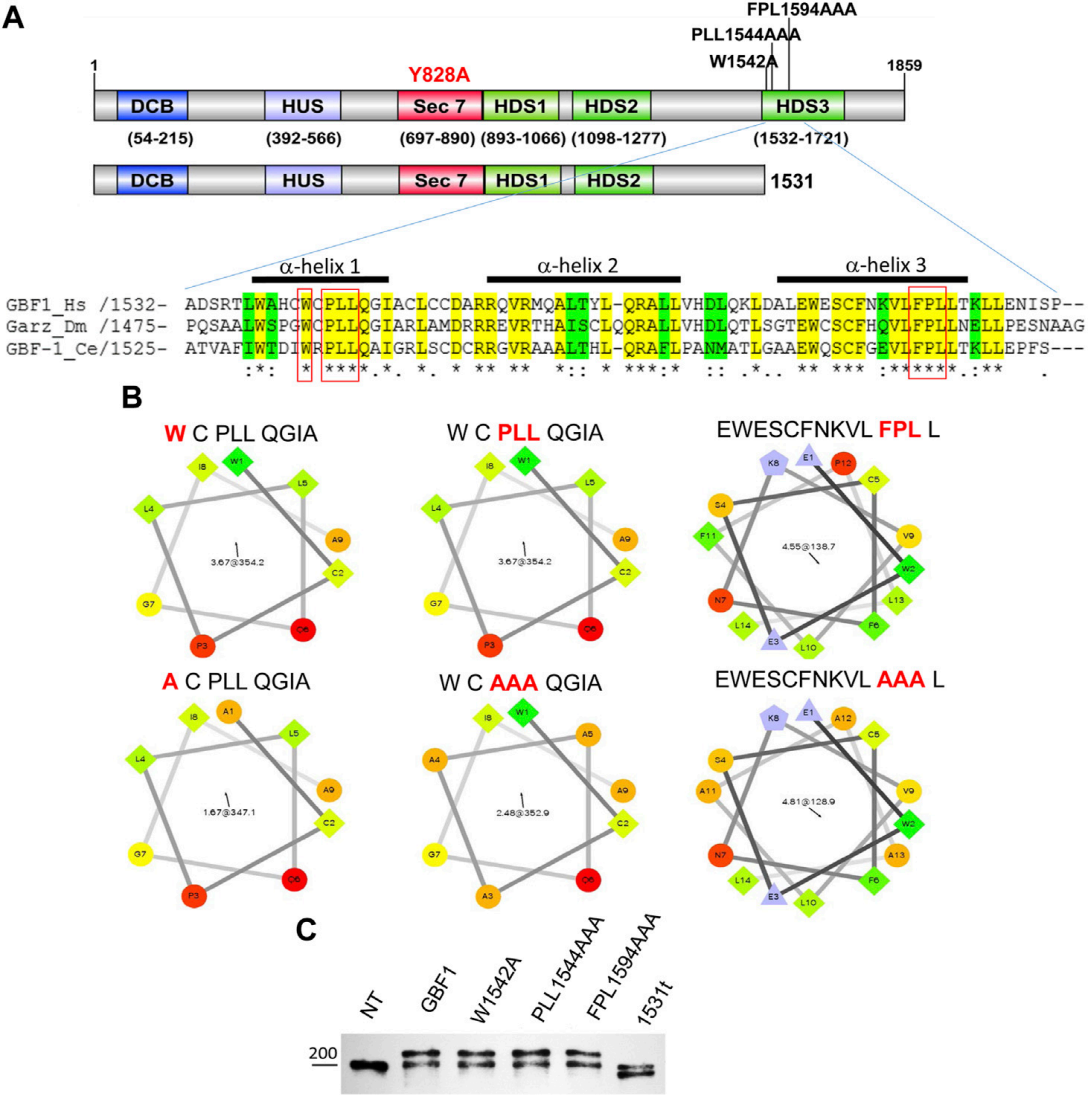
HDS3 mutants of GBF1 used in this study **(A)** A schematic representation of the domain structure of GBF1 showing the positions of the HDS3 mutations and the 1531 truncation (1531t construct). Alignment of the N-terminus of the HDS3 domains of GBF1 orthologs showing identical amino acids in yellow and conserved amino acids in green. The amino acids that were mutated to alanine in this study are boxed in red. The bars above the sequences indicate α-helices. (*Hs, Homo sapiens; Dm, Drosophila melanogaster; Ce, Caenorhabditis elegans*). **(B)** Helical wheel projections of α-helices analyzed in this study. Hydrophilic residues are circles, hydrophobic residues are squares, potentially negatively charged residues are triangles, and potentially positively charged residues are pentagons. The most hydrophobic residues are green, and the amount of green is decreasing proportionally to the hydrophobicity, with zero hydrophobicity coded as yellow. Hydrophilic residues are red with pure red being the most hydrophilic (uncharged) residue, and the amount of red decreasing proportionally to the hydrophilicity **(C)** Lysates from not transfected (NT) HeLa cells or cells expressing GFP-tagged GBF1 (GBF1) or the indicated HDS3 mutant for 24 h were resolved by SDS-PAGE and blotted with anti-GBF1 antibody. The endogenous GBF1 migrates at ∼204 kDa and represents a loading control in these experiments. The exogenous GFP-tagged GBF1 and the HDS3 mutants migrate at ∼230 kDa, while the GFP-1531t migrates at ∼196 kDa.

GBF1 is a large, multi-domain protein of ∼206 kDa (a schematic of domain arrangement is provided in Figure 1A). In addition to the centrally located ARF GEF catalytic Sec7d, it also contains the N-terminal dimerization and cyclophilin binding (DCB) and the homology upstream of Sec7 (HUS) domains, as well as three C-terminal homology downstream of Sec7 (HDS1-3) domains (Bui et al., 2009; Wright et al., 2014). This domain architecture has been preserved throughout evolution, and all GBF1 orthologs show analogous domain organization (Pipaliya et al., 2019). Moreover, the amino acid sequences within each domain of GBF1 orthologs are also conserved (Pipaliya et al., 2019).

Each domain of GBF1, except HDS3 has been shown to be essential for GBF1 function in cells, suggesting that each participates in processes that ensure the ability of the catalytic Sec7d to facilitate Arf activation on the membrane. The importance of the N-terminal domains has been documented by the cellular phenotypes caused by GBF1 mutations (truncation as well as point mutations) in these domains. For example, the loss of the DCB domain in the yeast ortholog of GBF1, Gea1p, adversely affects yeast viability (Spang et al., 2001). The DCB region of mammalian GBF1 (amino acids 1-215) also appears to be functionally important, as deletion of amino acids 1–294 results in loss of membrane association, which leads to inability to carry out its role at the Golgi (Mansour et al., 1998). Indeed, the very N-terminus is a critical region, as deleting or mutating only the N-terminal 10 amino acids of GBF1 to alanines totally inhibits its function (Viktorova et al., 2019). The HUS domain also is important as mutations within this domain (E646G, F481L, F477S, D485G, or F477S) in the yeast GBF1 ortholog Gea2p decrease its membrane association (Peyroche and Jackson, 2001; Park et al., 2005). GBF1 has been shown to be a dimer formed by the binding of the DCB domain to the HUS domain in the “opposite” monomer (Ramaen et al., 2007). Within the HUS domain, a highly conserved ∼9 amino acids “HUS” box is essential for the interaction with the DCB domain (Ramaen et al., 2007). Yet, it is unlikely that the mutations in DCB and HUS described above compromise GBF1/Gea1/2p function by affecting dimerization, because mutations within the DCB domain of GBF1 that prevent HUS binding and dimerization (K91A/E130A) do not affect GBF1 membrane association or GBF1 function in Golgi homeostasis and secretion (Ramaen et al., 2007; Bhatt et al., 2016). It is likely that the DCB/HUS mutations that reduce GBF1/Gea1/2p fitness do so by interfering with their functions independent of dimerization.

Both HDS1 and HDS2 domains are thought to be directly involved in membrane association. The HDS1 domain, immediately downstream from the Sec7d, is critical to GBF1 function, as mutations within this region (LF926,927AA) inhibit GBF1 membrane association by preventing GBF1 binding to a specific subset of PIPs [PI3P, PI4P and PI(4,5)P_2_] (Meissner et al., 2018). Thus, HDS1 may regulate GBF1 function in a manner similar to the PIP-binding PH domain found downstream of the Sec7d in other ARF GEFs (Stahelin et al., 2014). The HDS2 domain is also critical, as mutations of conserved residues in α-helix 2 (RDR1168/AAA) or α-helix 6 (LF1266/AA) prevent GBF1 association with Golgi membranes, inhibit GBF1 function in Golgi homeostasis and protein secretion, and lead to decreased cellular viability (Pocognoni et al., 2018). In support of the importance of HDS2 to GBF1 function, mutations (L1246/R; corresponding to L1250 in the human GBF1) in HDS2 of the *Danio rerio* zebrafish GBF1 cause disruption of vascular integrity leading to hemorrhage and embryonic death (Chen et al., 2017). In agreement, HDS1 and HDS2 domains in Gea1p and Gea2p are required for the localization of these GEFs to Golgi cisternae and their function, as their deletion causes cytosolic distribution and leads to decreased yeast cell viability (Gustafson and Fromme, 2017).

The role of the HDS3 domain in GBF1 function remains poorly understood. While the deletion of HDS3 appears to be inconsequential for membrane association and function of Gea1p and Gea2p (Gustafson and Fromme, 2017), an EMS-induced mutation in the *Drosophila melanogaster* fly ortholog of GBF1 called Gartzenwerg Garz^EMS667^ that introduces a frame shift at amino acid 1529 at the very beginning of the HDS3 domain leads to heart malformation and larval lethality (Wang et al., 2012). The HDS3 domain of GBF1 spans amino acids 1532–1721 and contains 7 predicted α-helices that exhibit stretches of amino acids showing absolute sequence conservation between species as diverse as humans, plants and yeast (Mouratou et al., 2005). Such strong level of evolutionary conservation is often indicative of functional importance. In this study, we either removed the HDS3 domain or targeted highly conserved residues within α-helices 1 and 3 to assess the role of HDS3 in GBF1 function. We show that the HDS3 mutants are compromised in Golgi binding and are unable to sustain Golgi homeostasis or secretion. Moreover, the critical role of the HDS3 domain in organismal homeostasis was documented by showing abnormal salivary gland and trachea development of mutant *Drosophila melanogaster* expressing Garz^EMS667^ lacking the HDS3 domain.

GBF1 exists in both cytosolic and membrane-bound pools and rapidly cycles between the cytosol and Golgi membranes (Niu et al., 2004; Szul et al., 2005). The diminished membrane association of GBF1 mutants with amino acid substitutions in DCB, HUS, HDS1, and HDS2 domains suggests that membrane binding involves a complex interplay of distinct domains to generate a binding interface and/or that multiple domains simultaneously engage the membrane. To gain insight into the domain organization that facilitates GBF1 interactions with the membranes, we analyzed the overall architecture of cytosolic and membrane-bound GBF1. We document that membrane binding induces dramatic rearrangements in GBF1, as shown by the exposure of multiple protease accessible sites that are masked in cytosolic GBF1. Importantly, extensive conformational changes were detected within regions bracketing the catalytic Sec7 domain, suggesting that its position relative to other domains is altered by GBF1 binding the membrane bilayer.

To provide structural information on domain organization within GBF1, we used negative staining electron microscopy to probe the architecture of full-length human GBF1. This analysis was performed on purified cytosolic GBF1 dimers. Obtained images were corelated with the AlphaFold predicted structure of GBF1 monomer to show that GBF1 is an anti-parallel dimer with a central core formed by the binding of the DCB-HUS domains from each monomer, and two arms composed of HDS1-3 domains protruding in opposite directions from the core. The two Sec7 domains extend in the opposite directions from the core of the dimer, and appear largely independent of the DSB-HUS domains. Our structural information on cytosolic GBF1 was combined with our biochemical data reporting on domain rearrangements within GBF1 upon membrane binding to inform parameters of GBF1 orientation required for Arf activation at the Golgi.

## Materials and methods

### Reagents

Brefeldin A (Sigma- Aldrich, MO, United States); Pierce Glutathione Agarose (Thermo Scientific, IL, United States); Tween-20 (Fisher Scientific, United States); SuperSignal^®^ West Femto Maximum Sensitivity Substrate (Thermo Scientific, IL, United States); Complete Protease Inhibitors Cocktail, EDTA-free (Santa Cruz, CA, United States); 8% and 10% PAGE gels and molecular weight standards were purchased from Invitrogen, Madison, United States; BODIPY TR (Texas Red) C5-Ceramide complexed with BSA (ThermoFisher scientific, Grand Island, NY), EnduRen cell permeable Renilla luciferase substrate (Promega, Madison, WI, United States).

### Antibodies

Polyclonal and monoclonal GFP antibodies (Abcam, MA, United States; catalog numbers ab290 and ab12091, respectively); Monoclonal GBF1 (raised against aa 1266-1379) antibodies (Santa Cruz, CA, United States; catalog number: sc-136240); Monoclonal GM130 (BD Transduction Laboratories, ON, United States; catalog number: 610823); Polyclonal GM130 (Thermo Scientific, IL, United States; catalog number: PA1-077); Monoclonal anti-Crumbs (Developmental Studies Hybridoma Bank (DSHB), IO, United States; catalog number Cq4); Monoclonal anti-GAPDH (Abcam, MA, United States; catalog number Ab8245); Rabbit polyclonal anti-calnexin (Enzo, NY, United States; catalog number: ADI-SPA-860-D); Monoclonal anti-GLUC (New England Biolabs; catalog number E8030); Monoclonal anti-β actin-HRP conjugated (Millipore Sigma; catalog number A3854); and polyclonal anti-myc (Abcam, MA, United States; catalog numbers ab9106). For immunofluorescence, anti-GFP were used at 1:500; anti-GM130 (BD Transduction) were used at 1:300; and anti-Crumbs were used at 1:50. Secondary antibodies conjugated with Alexa 488 Plus or Alexa 594 Plus (Invitrogen, Wi, United States and Thermo Scientific, IL, United States; catalog numbers: A32731 and A32740 respectively) were used at 1:500. For immunoblotting, rabbit anti-GFP were used at 1:1000; anti-GLUC were used at 1:1000; anti-β actin were used at 1:10,000; anti-calnexin were used at 1:1000; anti-GAPDH were used at 1:4000; anti-myc were used at 1:2,000; and anti-GBF1 were used at 1:200.

Secondary, anti-rabbit and anti-mouse antibodies conjugated with HRP (Pierce/Thermo Fisher Scientific Inc., IL, United States and Southern Biotech, AL, United States; catalog numbers: 31460 and 1030-05, respectively) were used at 1:1500.

### Mammalian cell culture and transfection

Human HeLa (CCL-2) cell line was obtained from ATCC, The Global Bioresource Center, United States. Human HEK (GripTite™ 293 MSR, R79507) cell line was purchased from ThermoFisher scientific, NY, United States. Cells were cultured in Minimum Essential Medium Eagle (Cellgro, Manassas, VA, United States) supplemented with 10% fetal bovine serum, 100 units/mL penicillin, 100 mg/mL streptomycin, and 1 mM sodium pyruvate and essential amino acids (Cellgro Manassas, VA, United States) at 37°C in humidified atmosphere. Cells were transfected with Mirus TransIT-LT1 Transfection Reagent (Mirus Bio Corporation, Madison, WI), according to manufacturer’s instructions.

### Plasmids

All HDS3 mutations were introduced into the Y828A GBF1 mutant described previously (Boal et al., 2010) (in some experiments, the A795E plasmid described previously (Belov et al., 2008; Belov et al., 2010) was the template) using the QuikChange XL Site-directed mutagenesis kit from Agilent Technology. The NH2-terminal mammalian pCMV-GST vector was a generous gift from Dr. R. Reed (Tsai and Reed, 1997). The PCR template was the pdt-Tomato-tagged- human GBF1 and the reactions were ran using Phusion Hot Start II High-Fidelity polymerase from Thermo-Scientific. The PCR products were cloned into the pCMV-GST vector with XhoI and NotI restriction enzymes (Promega). The sequence of the plasmid was confirmed by the UAB Genomics Core. The sequences of the oligonucleotide primers used for PCR were: Forward primer (5 = -GAG​CCT​CGA​GCG​ATG​GTG​GAT​AAG​AAT-3) and Reverse primer (5 = − GAT​AGC​GGC​CGC​TTA​GTT​GAC​CTC​AGA​GGT-3).

### Sequence alignments

Protein sequences for *Homo sapiens (Hs)* GBF1 and orthologs from *Drosophila melanogaster (Dm) and Caenorhabditis elegans (Ce)* were retrieved from GenBank and HDS3 domains were aligned using Clustal Omega (Lin et al., 2005; Simossis et al., 2005; Simossis and Heringa, 2005) alignments (Li et al., 2015; Squizzato et al., 2015).

### Immunofluorescence and confocal microscopy

HeLa cells were seeded overnight on glass coverslips (ø12 mm), transfected, and 24 later processed for IF as described in (Bhatt et al., 2019). For BFA replacement assay, transfected cells were washed in phosphate-buffered saline (PBS) and incubated with 0.5 μg/mL BFA for 30 min before fixation and processing for IF. Cells were visualized under a Leitz Wetlzar microscope with epifluorescence and Hoffman Modulation Contrast optics by Chroma Technology, Inc. (Bellows Falls, VT, United States). Images were captured with a 12-bit CCD camera from QImaging (Surrey, BC, Canada) and processed with iVision-Mac software. For all experiments involving quantification, imaging was performed on cells from at least 2 independent transfections.

Confocal imaging was with Perkin Elmer Ultraview ERS 6FE spinning disk confocal attached to a Nikon TE 2000-U microscope. The system was equipped with laser and filter sets to visualized and image FITC, TRITC and DAPI fluorescence. Images were captured using a Hamamatsu C9100-50 EMCCD camera (Hamamatsu Photonics K.K., Hamamatsu city, Japan) and 60X or ×100 Plan APO oil-immersion objectives. The imaging system was operated by Volocity 6.2 software (Perkin Elmer, Shelton, CT, United States).

### Live cell imaging

Constructs were transfected into HeLa cells on coverslips for 16 h. Coverslips were washed three times with an imaging medium buffered with HEPES, pH 7.4 (Live Cell Imaging solution- Molecular Probes, Grand Island, NY). Cells were incubated with 1 µM BODIPY TR (Texas red) C5-Ceramide complexed with BSA (ThermoFisher scientific, Grand Island, NY) for 30 min at 37°C to label the Golgi apparatus, washed five times with HEPES imaging media and incubated in fresh MEM medium at 37°C for 30 min. Live cell imaging was performed using confocal microscopy as described above. The coverslips were maintained on a thermostage at 37°C, 5% CO2 and 70% relative humidity in HEPES imaging media.

Images were analyzed by Volocity software. To identify the ceramide stained Golgi region (red), the threshold in the red channel was set to the average intensity across the entire image +5 standard deviations. All the identified Golgi regions were verified by visual inspection. The outer boundary of cells was delineated by using the average green intensity across the entire image. The total intensity of green within the cell (total cellular GFP-GBF1 levels) and the intensity of green that colocalizes with red (Golgi-resident GFP-GBF1) was then determined and the % of total GBF1 that resides at the Golgi was calculated. Values were obtained from two independent transfections.

### Western blotting

Cells were lysed in RIPA buffer (50 mM Tris-HCl, pH 7.5, 150 mM NaCl, 1% Nonidet P-40, 0.5% Deoxycholate Na, 0.1% SDS, and fresh protease inhibitors cocktail) or HKMT buffer (20 mM Hepes, pH 7.4, 0.1 M KCl, 1 mM MgCl_2_, 0.5% Triton X-100, and fresh protease inhibitors cocktail). Resolved gels were transferred to nitrocellulose membrane (GE Healthcare, Piscataway, NJ, United States) using Biorad mini protean tetra cell (Bio-Rad Laboratories Inc., Hercules, California, United States). Transfer was followed by blocking with 5% milk in PBS with 1% Tween 20 (PBST) for 1 h. Membranes were probed with the indicated primary antibodies for 1 h, washed with PBST and incubated with horse-radish peroxidase conjugated anti-rabbit or anti-mouse secondary antibody for 1 h followed by washing with PBST and developed with SuperSignal^®^ West Femto Maximum Sensitivity Substrate (Thermo Scientific, Rockford, IL, United States) and exposing the membranes to X-ray films.

### Luciferase secretion assay

HeLa cells were co-transfected with pCMV-GLUC encoding Gaussia luciferase and either an empty vector or plasmids coding for the BFA-resistant wild-type or mutant GBF1 plasmids and processed as in (Bhatt et al., 2019). Samples were quantitated using a Tecan M1000 multifunctional plate reader. Signal from at least 8 wells was averaged for every sample.

### Cell fractionation and limited proteolysis

HeLa cells transfected with GFP-GBF1 constructs for 24 h were fractionated as in (Szul et al., 2005). Briefly, cells were washed with PBS and disrupted in 300 mL of homogenization buffer (50 mM HEPES–KOH, pH 7.5, 100 mM KCl, 1 mM MgCl2, 1 mM DTT) containing protease inhibitors by repeated passage through a 27G needle. The homogenate was centrifuged at 1000xg for 15min at 40C in a microcentrifuge to remove unbroken cells and nuclei. The postnuclear supernatant was centrifuged in an ultracentrifuge at 100,000xg for 60min at 4°C in a Beckman TLA 100.2 rotor. The supernatant fraction was designated cytosol fraction, and the pellet was rinsed once with homogenization buffer and recentrifuged under the same conditions. The resulting pellet was solubilized in RIPA buffer with protease inhibitors and designated membrane fraction.

In some experiments, fractions containing the same volume of original cells were analyzed by 8% SDS–PAGE, and the separated proteins were transferred to NitroPure nitrocellulose (NC) membrane (Micron Separations, Westborough, MA, United States). The NC membrane was cut into three sections and immune-blotted with anti-GFP, anti-Calnexin and anti-GAPDH antibodies as previously described (Zhang, 2016).

In some experiments, cytosol and membrane samples (equal amount of protein in 20 mM Hepes pH 7.4, 0.1 M KCl, 1 mM MgCl2) were treated with 2.5 μg/mL of porcine trypsin in 50 mM Tris-HCl, pH 8.0, 50 mM NaCl, 1 mM CaCl2. Digested samples at time points 15 and 30 min were analyzed by running on an SDS-PAGE gel and immunoblotting with indicated antibodies (marked in Figures) as described above. A standard migration curve was plotted on a logarithmic graph using the migration distance of the Molecular weight ladder bands. The approximate size (in kDa) of the cleaved fragments were estimated by plotting their migration distance on the standard curve. We determined the possible trypsin cleavage sites in the sequence of human GBF1 by using “Peptide Cutter” tool on the ExPASy database.

### Drosophila melanogaster embryos development

Flies were kept at 25°C under standard conditions. The *garz* mutant *garz* ^*EMS667*^ was kindly provided by Drs. Marcus Affolter and Achim Paululat (Wang et al., 2012). For distinguishing homozygous and heterozygous embryos *garz* ^*EMS667*^ allele was balanced over *CyO, dfd-eYFP* (Le, Liang, Patel et al., Genetics, 2006). Homozygous embryos were identified by the absence of eYFP fluorescence. Embryos were staged according to (Campos-Ortega and Hartenstein, 1985). For salivary gland immunostaining, *garz* ^*EMS667*^ */garz* ^*EMS667*^ and *garz* ^*EMS667*^ */CyO,dfd-eYFP* (control) embryos at stage 15 were manually dechorionized and devitellinized, fixed in 4% formaldehyde in PBS, permeabilized with PBST (PBS supplemented with 0.2% Triton X-100) and blocked with PBST containing 10% goat serum. Embryos were incubated with antibodies against Crumbs at 4°C. The primary antibodies were detected by 2-h incubation at room temperature with secondary antibodies conjugated to Alexa488 or Alexa568 (Molecular Probes^®^, 1:300). Nuclei were counterstained with DAPI (Roche) and slides were mounted with Fluoromount-G^®^ (SouthernBiotech). Images were taken with an Olympus BX61 motorized upright microscope fitted with a BX-DSU disc scan unit. For trachea development experiment, *garz* ^*EMS667*^ */garz* ^*EMS667*^ and *garz* ^*EMS667*^ */CyO,dfd-eYFP* (control) embryos at stage 16–17 were manually dechorionized, placed on a slide covered with halocarbon oil and examined under a bright field. Live images were taken using Olympus MVX10 MacroView microscope.

### Purification of GST-GBF1

HEK cells were transfected with GST-GBF1 plasmid and 24 h later were lysed in lysis buffer (50 mM Bis-Tris, pH 7.5, 150 mM NaCl, 1 mM DTT, protease inhibitors cocktail ThermoFisher Scientific, cat. A32955). Lysate was passed 5-7 times through a 23-gauge needle, then through a 27-gauge needle and centrifuged at 4°C at 1000 RCF for 15 min. The supernatant was incubated with glutathione agarose beads (ThermoFisher Scientific, cat. 16100) at 4°C for 4 h. Beads were washed three times with wash buffer (20 mM Bis-Tris, pH 7.0, 200 mM NaCl, 1 mM DTT) and eluted with elution buffer (wash buffer with the addition of 15 mM reduced glutathione). Eluted protein was further purified on HiTrap Q HP anion exchange column (Cytiva, cat. 17115301) using a gradient of 100 mM–1M NaCl in 20 mM Bis-Tris, pH 7.0, 1 mM DTT.

### Electron microscopy

A sample (3 μL) of purified GST-GBF1 was applied to glow-discharged 400 mesh copper grids with an ultrathin continuous carbon substrate over a lacey carbon support film (Electron Microscopy Sciences, Hatfield, PA), washed with low salt buffer (20 mM Bis-Tris, pH 7.0, 50 mM NaCl, 1 mM DTT), and negatively stained with 1% uranyl formate (Polysciences, Inc. Warrington, PA, United States). The grid was imaged in an FEI Tecnai F20 electron microscope (Eindhoven, Netherlands) operated at 200 kV and a magnification of ×34,700 (1.43 Å/pix) with a Gatan K3 direct electron detector (Pleasanton, CA). 20-frame movies were semi-autonomously collected with a defocus range of −1.25 μm to −2 μm and motion-corrected in SerialEM (Mastronarde, 2005).

### Single particle analysis

Micrographs were imported to RELION-3.1.0 for single particle analysis (Zivanov et al., 2018). CTFs were estimated with Gctf (Zhang, 2016), and particles were initially picked with Topaz using the default resnet8_u64 model with 4x binning and a radius of 16pix (Bepler et al., 2019). Iterative 2D classification produced classes depicting the expected structure of GBF1, which were used for a second round of template-based autopicking using the RELION-3.1.0 autopicker. The final 2D classes were generated from 81,522 particles without binning.

## Results

### GBF1 function in Golgi homeostasis and secretion requires intact HDS3

The importance of the C-terminal HDS3 domain to GBF1 localization, stability, and function in cells was assessed by comparing wild-type and four different GBF1 mutants with amino acid substitutions within HDS3. We generated a truncated GBF1, lacking all of HDS3, by introducing a stop codon after residue 1531 (1531t), and three missense mutants that alter the most evolutionarily conserved residues in α-helix 1 and α-helix 3 to alanine; W1542A (W), PLL1544AAA (PLL) and FPL1594AAA (FPL) (Figure 1A, residues boxed in red). The substitution of the hydrophobic W, L or F residues with Alanine, carrying a smaller side chain, alters the surface characteristics of each helix (Figure 1B). In this study, each construct is tagged at the N-terminus with GFP (except when stated), and all constructs contain the Y828A mutation within the catalytic Sec7d that confers BFA resistance to human GBF1 (Boal et al., 2010). This latter mutation allows functional testing of the exogenously expressed proteins in cells in which the endogenous GBF1 is inactivated with BFA. The truncation or mutations in the HDS3 domain had no detectable effect on the protein’s expression or stability, as equivalent levels of all proteins were detected when transiently expressed in HeLa cells (Figure 1C; Western immunoblotted with anti-GBF1 to detect the endogenous GBF1 at ∼204 kDa and the recombinant GFP-tagged proteins at ∼234 kDa. Note that the GFP-GBF1/1531t construct, predicted to be ∼196 kDa, migrates slightly below the endogenous GBF1 that runs near the 200 kDa marker, as expected). The levels of the recombinant proteins appear very similar to the endogenous GBF1, and considering that the transfection efficiency in our experiments is ∼70% (data not shown), suggest that the recombinant constructs are expressed within cells at ∼1–1.5 fold the levels of the endogenous GBF1.

**FIGURE 2:**
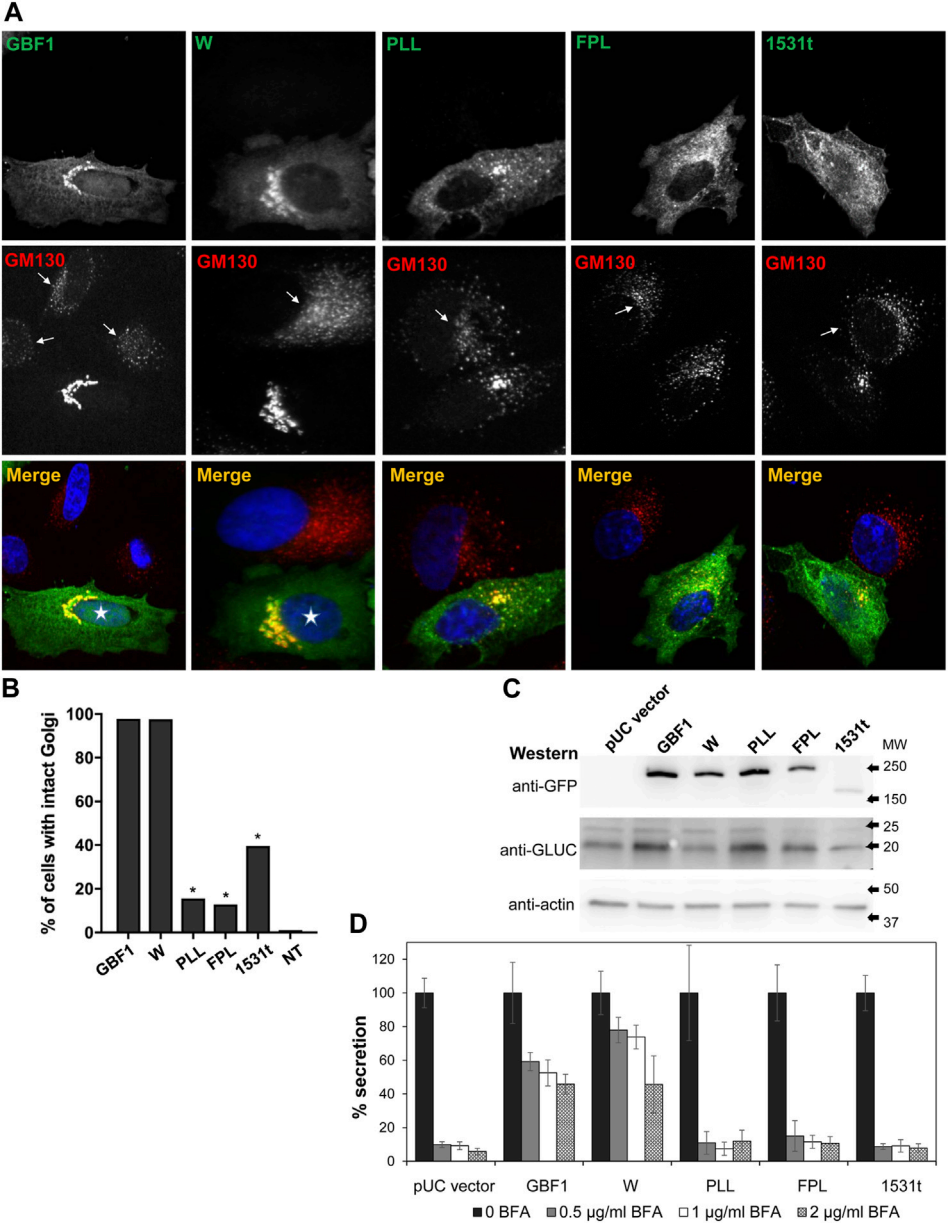
HDS3 mutants are defective in supporting Golgi architecture and secretion **(A)** HeLa cells were transfected with the indicated GFP-tagged, BFA-resistant construct. After 24 h, cells were treated with 0.5 μg/mL BFA for 30 min and then processed for IF with anti-GFP and anti-GM130. Cells expressing GBF1 or the W mutant maintain intact Golgi in the presence of BFA (asterisks in merged panels), while cells expressing the PLL, FPL and the 1531t constructs have disrupted Golgi. Untransfected cells show disrupted Golgi (arrows in GM130 panels). **(B)** Quantification of Golgi phenotype in HeLa cells treated as in A. Golgi structures were scored in transfected cells and the results are presented as the % of transfected cells with intact Golgi. In the presence of 0.5 μg/mL of BFA, 97.7% of GBF1 (*n* = 45), 97.5% of the W mutant (*n* = 40), 15.6% of the PLL mutant, 12.9% of the FPL mutant (*n* = 31), and 39.5% of the 1531t mutant (*n* = 30) had intact Golgi. (* = *p* < 0.05, Chi square statistic). **(C)** HeLa cells were transfected with pGLuc alone (pUC vector) or were co-transfected with pGLuc and the indicated GFP-tagged GBF1 construct. After 24 h, cells were lysed and the lysates were analyzed by SDS-PAGE followed by transfer to a nitrocellulose filter. Each filter was cut into 3 regions spanning different range of molecular sizes, and each piece was immunoblotted with the indicated antibodies. Actin represents the loading control. GLUC and a GBF1 protein is expressed in all cells. **(D)** HeLa cells were transfected with pGLuc alone (pUC vector) or were co-transfected with pGLuc and the indicated BFA-resistant GFP-tagged GBF1 construct. After 24 h, fresh media containing the indicated concentration of BFA were added. After 4 h, levels of luciferase in medium were measured and are presented as % of luciferase secreted in the absence of BFA for each construct.

To assess the functionality of the GBF1 constructs, we first confirmed that cells expressing the BFA-resistant GFP-GBF1 and treated with 0.5 μg/mL of BFA for 30 min maintain normal Golgi structure, as shown by the characteristic distribution of the Golgi marker GM130 (Figure 2A, transfected cells marked with asterisk in merged panel), while untransfected cells show dispersed Golgi, a characteristic of inhibition of endogenous GBF1 by BFA (Figure 2A, arrows in GM130 panel point to cells not expressing exogenous proteins). The ability of the HDS3 mutants to support Golgi architecture was tested next. While the W1542A mutant was functional, resulting in normal appearing Golgi, the PLL1544AAA, FPL1594AAA, and 1531t mutants were each unable to maintain the characteristic, ribbon-shaped Golgi, with the transfected cells showing Golgi dispersion comparable to untransfected cells treated with BFA (Figure 2A). Quantification of the phenotypes revealed that the GBF1 and the W1542A mutant support Golgi architecture in >97% of cells in which they are expressed, while the PLL1544AAA, FPL1594AAA, and 1531t constructs maintain intact Golgi in only ∼10–40% of transfected cells (Figure 2B).

To assess the ability of the HDS3 mutants to support secretion, we used an assay based on the secretion of Gaussia luciferase (GLUC) with a natural secretion signal that targets it into the secretory pathway in human cells (Tannous et al., 2005). In this assay, pGLuc plasmid encoding GLUC is transfected alone or is co-transfected with a GBF1 construct into HeLa cells, and after 24 h, cells are supplemented with fresh media containing different concentrations of BFA to inactivate endogenous GBF1, and secretion of GLUC into the medium is measured over the subsequent 4 h. The rationale for this assay is that secretion will occur only if the exogenously expressed GBF1 is functional, but GLUC will not be secreted if the exogenously expressed GBF1 is functionally compromised.

To validate the assay and ensure that co-transfection of HeLa cells with pGLuc and GBF1 constructs results in the expression of both proteins, pGLuc alone or pGLuc with each GBF1 construct was co-transfected into HeLa cells, and the expression of the corresponding proteins was assessed 24 h later by Western blotting. As shown in Figure 2C, GLUC, as well as wild-type and mutant GBF1 are detected in each lysate. To determine the ability of each GBF1 mutant to sustain secretion, pGLuc alone or with each GBF1 construct was co-transfected into HeLa cells, and after 24 h, cells were incubated in fresh media containing different concentrations of BFA, and secretion of luciferase into the medium was measured over the subsequent 4 h. As shown in Figure 2D pUC vector graph, cells expressing only the pGLuc construct secrete luciferase in the absence of BFA, but secretion is inhibited by even the lowest concentrations of BFA. This reflects the inhibition of the endogenous GBF1 by BFA. In contrast, cells expressing the BFA-resistant GBF1 secrete significant levels of luciferase (∼60–50% of that without BFA) even at the highest BFA concentrations. This represents the highest standard for effective rescue from BFA inhibition by a functional GBF1. Cells expressing the W1542A mutant also efficiently secrete luciferase in the absence and in the presence of BFA. In contrast, cells expressing the PLL1544AAA, FPL1594AAA or 1531t mutants secrete luciferase in the absence of BFA, but are inhibited even at the lowest concentration of BFA (<10% secretion of that without BFA). Thus, a truncation that eliminates HDS3, or changes in conserved residues within the HDS3 (PLL1544AAA, FPL1594AAA) render GBF1 unable to support Golgi homeostasis and secretion, and imply that the HDS3 domain performs a critical role in GBF1 function.

### Intact HDS3 is required for GBF1 targeting to Golgi membranes

**FIGURE 3:**
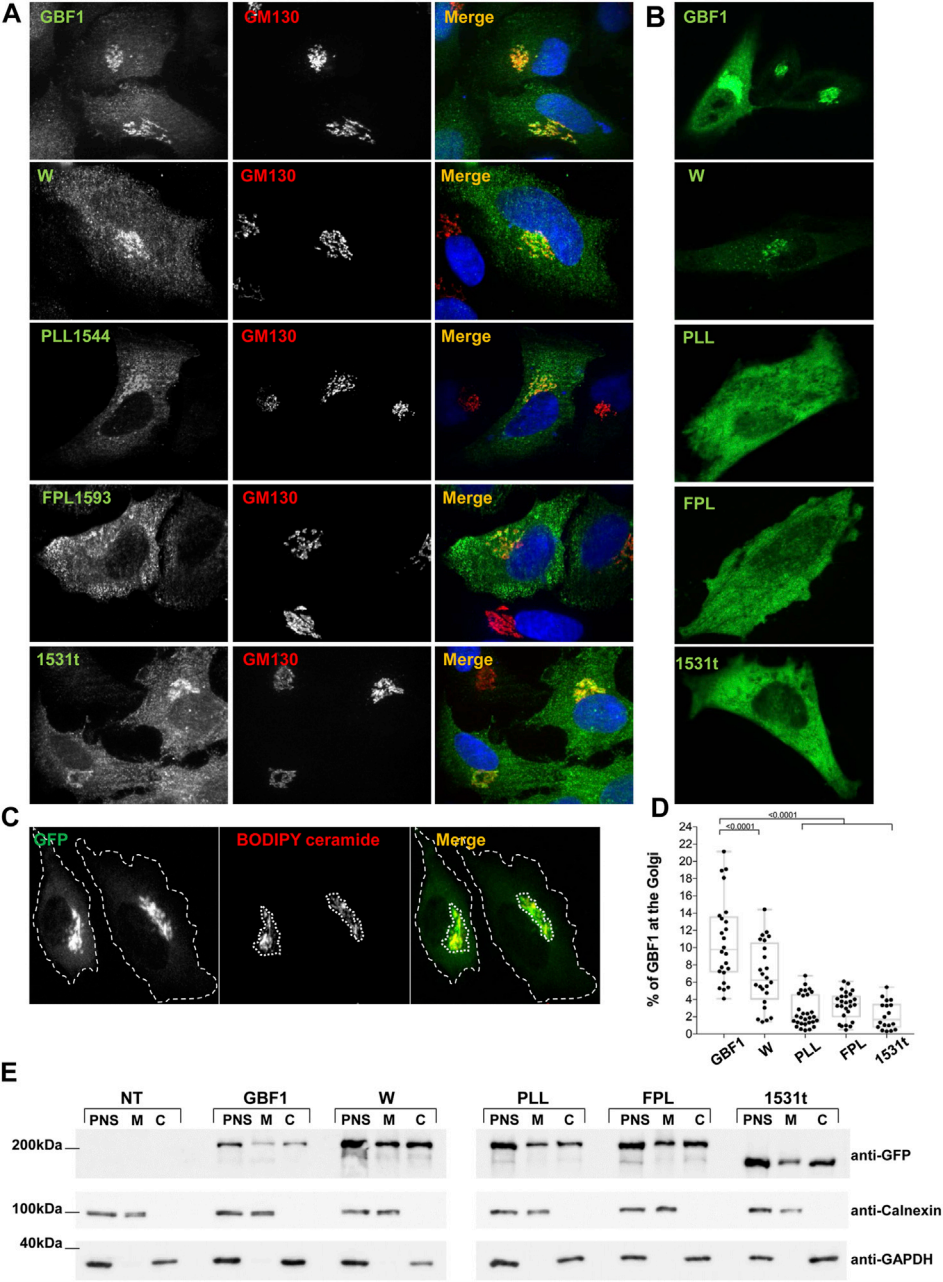
HDS3 mutants are defective in Golgi targeting **(A)** HeLa cells were transfected with GFP-tagged GBF1 (GBF1) or the indicated HDS3 mutant and after 16 h, processed for IF to detect the GFP tag and the GM130 Golgi marker. GBF1 and the W and 1531t mutants are readily detected at the Golgi, while the PLL and FPL mutants show a more dispersed distribution. **(B)**. HeLa cells were transfected with GFP-tagged GBF1 (GBF1) or the indicated HDS3 mutant and after 16 h were imaged under live-cell conditions. GBF1 and the W mutant are detected in morphologically recognizable Golgi structures. In contrast, the PLL, FPL and 1531t mutants show diffuse cellular distribution. **(C)** HeLa cells were transfected with GFP-tagged GBF1 or a HDS3 mutant and after 16 h stained with BODIPY TR C5-Ceramide for 30 min and then imaged under live-cell conditions. The outer boundary of the cells (green) and the BODIPY ceramide-stained Golgi region (red) were computationally determined as indicated by the dotted outlines (a representative staining of GFP-tagged GBF1 is shown). **(D)** HeLa cells were transfected with GFP-tagged GBF1 (GBF1) or the indicated HDS3 mutant and after 16 h stained with BODIPY TR C5-Ceramide for 30 min and then imaged under live-cell conditions. The outer boundary of the cells (green) and the BODIPY ceramide-stained Golgi region (red) were computationally determined and the % of green fluorescence that resides at the Golgi was calculated for each construct. The values (% ± SEM) are: GBF1 = 10.8 ± 0.9 (*n* = 24 cells), W = 6.8 ± 0.8 (*n* = 24 cells), PLL = 2.6 ± 0.3 (*n* = 30 cells), FPL = 3.3 ± 0.3 (*n* = 26 cells), 1531t = 2.2 ± 0.4 (*n* = 18 cells). The Golgi targeting values for the HDS3 mutants are statistically significantly different as compared to GBF1. (*t*-test, * = *p* < 0.05). **(E)** HeLa cells were not transfected (NT) or transfected with GFP-tagged GBF1 (GBF1) or the indicated HDS3 mutant and after 24 h, cells were fractionated to generate the post-nuclear supernatant (PNS) that was subsequently fractionated into cytosol **(C)** and total membranes (M). Aliquots of each fraction were separated by SDS-PAGE and transferred to nitrocellulose. The filter was cut into 3 section and immunoblotted with anti-GFP to detect the GBF1 constructs, anti-calnexin to detect membranes, and anti-GAPDH to detect cytosol. All constructs can be detected in the membrane fractions, indicating that the mutants retain membrane-binding ability.

To probe the mechanistic basis of the functional defects exhibited by the PLL1544AAA, FPL1594AAA and 1531t constructs, we assessed their ability to target to the Golgi. Standard immunofluorescence localization of each construct relative to the cis-Golgi marker GM130 showed that the constructs associate with the Golgi to different extents (Figure 3A). In this analysis, we used fixed cells that have been permeabilized with Triton-X-100, which causes the loss of cytosolic proteins and preferentially showcases proteins associated with membranes. GBF1, the W1542A and the 1531t mutants were each predominantly detected in a morphologically recognizable Golgi; the PLL1544AAA mutant appears less able to target to the Golgi and the FPL1594AAA mutant shows minimal Golgi staining and is mostly in a peripheral haze (Figure 3A). To overcome the potential loss of the cytosolic pool of GBF1 due to cell permeabilization, we measured targeting efficiency in live cells: GBF1 and the W1542A mutant were readily detected at the Golgi, but the PLL1544AAA, FPL1594AAA, and 1531t mutants appeared diffuse within the cells, without a clearly defined Golgi pattern (Figure 3B). The amounts of each construct localizing to the Golgi were quantified in live cells after staining with the vital Golgi stain BODIPY TR C5-Ceramide (Figure 3C, representative images). The computational measurement of the outer boundary of cells (green channel) and the BODIPY ceramide-stained Golgi region (red channel) determined that ∼11% of exogenously expressed GBF1 associated with the Golgi, in agreement with results of previous fractionation experiments (Donaldson et al., 1992; Kahn et al., 1992; Szul et al., 2005; Szul et al., 2007) (Figure 3D). The W1542A mutant retained partial targeting ability, with ∼7% of total expressed protein localizing to the Golgi, while only 2%–3% of the PLL1544AAA, FPL1594AAA, and 1531t constructs localized to the Golgi. These data suggest that an intact HDS3 domain is a critical determinant of GBF1 localization to the Golgi.

This phenotype could be caused by the inability of the PLL, FPL, and 1531t mutant to target to the Golgi or a general inability to associate with membranes. To probe the basis of the lack of Golgi localization, we fractionated post nuclear supernatants (PNS) of cells expressing each construct into cytosol (C) and membranes (M) and assessed the recovery of each GBF1 mutant in the membrane fraction. As shown in Figure 3E, our fractionation protocol effectively separates membranes from cytosol, as shown by the differential recovery of calnexin (membrane marker) and GAPDH (cytosolic marker) in the corresponding fractions. Interestingly, all GBF1 constructs, including the PLL, FPL, and 1531t mutants that do not target to the Golgi retain the ability to associate with cellular membranes. These results suggest that an intact HDS3 is required for the selective targeting and the preferential engagement of GBF1 with the Golgi.

### HDS3 of Garz is required for *D. melanogaster* development

The sole ortholog of the mammalian GBF1 in the fruit fly *D. melanogaster* is termed Garz. To define the importance of an intact HDS3 domain within an organismal context, we assessed the consequences of eliminating HDS3 from Garz on its functionality in flies. Garz is continuously expressed during development, with highest abundance in glandular and tubular organs (Wang et al., 2012). Garz is essential for development, as flies deleted of Garz die early during embryogenesis (Armbruster and Luschnig, 2012). Moreover, we have shown that tissue- and stage-specific depletion of Garz from the salivary gland in the third instar larvae, at a time of maximal salivary gland development, causes disorganized lumen and decreases the growth of the gland (Szul et al., 2011).

**FIGURE 4:**
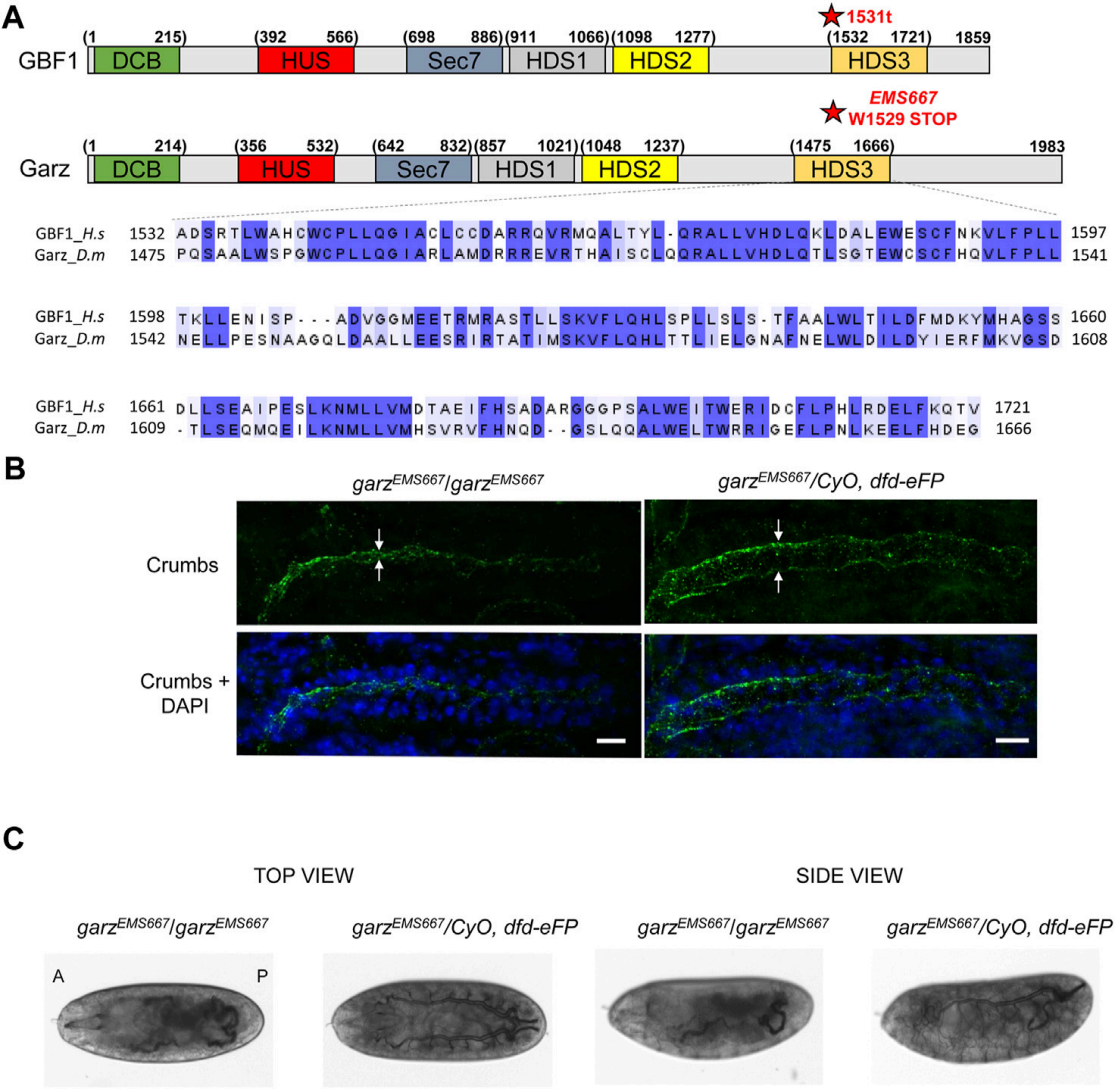
HDS3-containing region of Garz is required for *Drosophila* development **(A)** A schematic representation of human GBF1 and *Drosophila* Garz with asterisks marking the 1531t and the *EMS667* truncations. Sequence alignments show high level of conservation throughout the HDS3 domain. **(B)** Homozygous and heterozygous *EMS667 garz* allele embryos at stage 15 were analyzed for salivary gland development by staining with Crumbs marker of the apical membrane of salivary cells and DAPI to visualize epithelial cells. In homozygous *EMS667* embryos the diameter of the salivary gland lumen is decreased compared to the heterozygous control embryos. Scale bars: 10 μm. **(C)** Homozygous and heterozygous *EMS667 garz* allele embryos were analyzed for tracheal development at stage 17. Paired branched tracheal tubes filled with air are evident in the heterozygous control embryos, but are convoluted and disorganized in the *EMS667* homozygous embryos.

Alignment of the amino acid sequences of the HDS3 domains from human GBF1 and *D. melanogaster* Garz shows 54% identity and 70% similarity over the ∼200 amino acids-long domain (Figure 4A). Such high level of conservation suggested that like in human GBF1, HDS3 may be important for Garz function. To test this, we assessed organ development in flies expressing a truncated Garz, lacking the HDS3 domain. Fortuitously, a previous screen isolated an EMS-induced *garz* ^EMS667^ allele mapped to a single G to A mutation that results in a premature stop codon and generates a Garz protein truncated at amino acid 1529 near the N-terminus of the HDS3 domain (Figure 4A) (Wang et al., 2012). The truncation generates a Garz mutant protein similar to the dysfunctional mammalian GBF1 1531t mutant, and allows the analysis of Garz^EMS667^ function within a living organism. Specifically, we assessed tubulogenesis in the salivary glands and in the trachea of the *garz* ^EMS667^ mutant *Drosophila* embryos.

One of the hallmarks of proper salivary gland development is the expansion of the lumen during embryo growth (Kerman et al., 2006) that can be visualized by immunostaining for Crumbs, a transmembrane protein marker of the apical membrane of epithelial salivary cells (Tepass et al., 1990). As shown in Figure 4B, the diameter of the salivary gland lumen was greatly reduced in homozygous *garz* ^*EMS667*^ */garz* ^*EMS667*^ mutant embryos when compared to heterozygous control embryos, indicating abnormal growth and expansion of the epithelial cells and leading to defects in the overall size and morphology of the gland.

During embryogenesis, the bilateral tracheas develop as branched arrays on the ventral side of the embryo and are filled with matrix proteins, which are subsequently cleared and replaced with gas just before the embryo hatches (Tsarouhas et al., 2007). As shown in Figure 4C, heterozygous *garz* ^*EMS667*^ */CyO, dfd-eFP* control embryos exhibit a highly ordered branched pattern of tubular elements, with cleared lumens indicative of gas filling. In contrast, homozygous *garz* ^*EMS667*^ */garz* ^*EMS667*^ mutant embryos have disorganized tracheal architecture and are defective in matrix clearance as shown by a failure of the tracheal tubes to clear and gas-fill. Due to these developmental defects homozygous mutants do not hatch into larvae and die as embryos. Thus, Garz functionality, like that of mammalian GBF1, is critically dependent on an intact C-terminal HDS3 domain.

### GBF1 undergoes conformational shifts upon membrane binding

**FIGURE 5:**
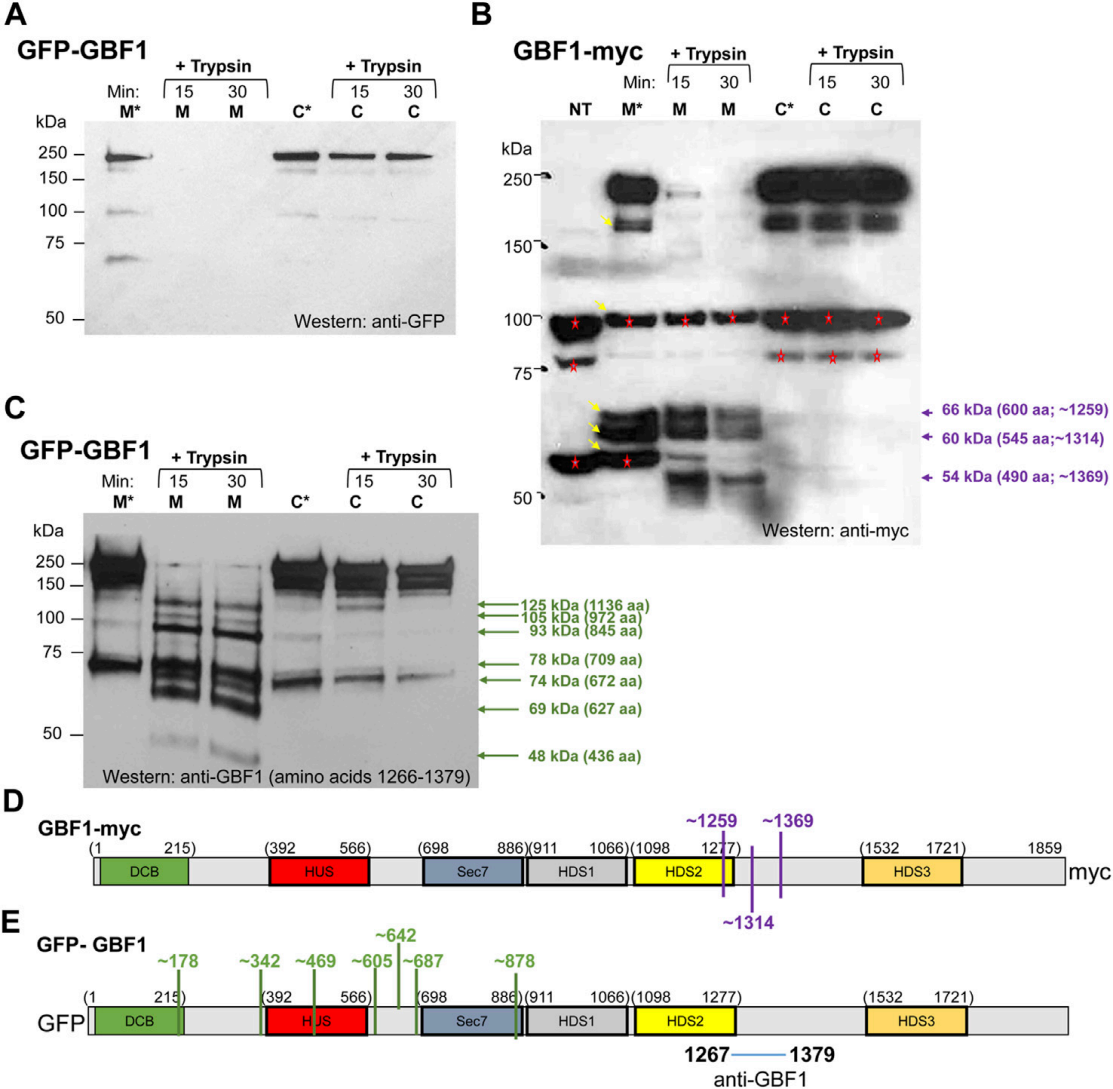
GBF1 undergoes conformational shifts upon membrane binding **(A–C)** HeLa cells were transfected with the indicated GBF1 construct (tagged with GFP at the N-terminus or with *myc* at the C-terminus) and after 24 h, cells were fractionated to generate a membrane (M) and a cytosol (C) fraction. Each fraction was incubated either with buffer alone for 30 min at 37°C (M* and C* lanes) or with buffer containing trypsin (+trypsin) for the indicated times at 4°C. Samples were analyzed by SDS-PAGE followed by Western blotting with the indicated antibodies (anti-GFP, anti-*myc* or anti-GBF1/1267-1379). **(B)** Bands recognized by anti-*myc* and not related to GBF1 are detected in the lysate from non-transfected cells (NT lane, bands marked with red asterisks). Such endogenous proteins recognized by anti-*myc* were also detected in other lanes (marked with red asterisks) and all were ignored during the subsequent analysis. GBF1 fragments generated in the membrane, but not the cytosol fraction are marked with violet arrows, together with the approximate MWs and number of amino acids within each fragment. **(D,E)** Approximated cleavage sites unmasked in the membrane-bound GBF1 calculated from fragments obtained by analyzing either the GBF1-*myc* cleavages from panel **(B)** (shown in D in purple) or the GFP-GBF1 cleavages from panel C (shown in E in green). The epitope recognized by the anti-GBF1/1267–1379 is contained within amino acids 1267–1379 and is marked. The cleavage sites were calculated based on amino acid 1314 being the C-terminus of each fragment.

GBF1 is a cytosolic protein that transiently associates with Golgi membranes to activate Arfs (Niu et al., 2004; Szul et al., 2005). Previous and this work documented that multiple domains of GBF1 are required for its membrane association and function. To provide insight into how GBF1 structure may change during membrane binding, we examined the overall architecture of cytosolic and membrane-bound GBF1 using limited tryptic proteolysis. Cells expressing an N-terminally GFP-tagged GBF1 (GFP-GBF1) were fractionated into cytosol and total membranes, and each fraction was either incubated at 37°C for 30 min or supplemented with trypsin and incubated at 4°C for different times. The samples were then analyzed by SDS-PAGE followed by Western blotting with an anti-GFP antibody. As shown in Figure 5A, full-length GFP-GBF1 was detected in the membrane (M* lane) and the cytosolic (C* lane) fractions incubated at 37°C without added trypsin (Figure 5A, M* and C* lanes). When trypsin was added to the membrane fraction for 15 or 30 min, GFP-GBF1 was no longer detected, indicating at least partial degradation (Figure 5A, M lanes). In contrast, full-length GFP-GBF1 was detected in the cytosolic fractions treated with trypsin for 15 or 30 min (Figure 5A, C lanes). The degradation of GFP-GBF1 in the membrane fraction but GFP-GBF1 persistence in the cytosolic fraction in the presence of trypsin suggests that membrane binding imposes a conformational shift that exposes cleavage site(s) that are protected within cytosolic GFP-GBF1.

To confirm the distinct conformations of the membrane associated and cytosolic GBF1, we compared the trypsin sensitivity of membrane-bound and cytosolic GBF1 tagged at the C-terminus with the *myc* epitope. Cells expressing GBF1 tagged at the C-terminus with the *myc* epitope (GBF1-*myc*) were fractionated into cytosol and total membranes, and each fraction was either incubated at 37°C for 30 min or supplemented with trypsin and incubated at 4°C for different times. The samples were then analyzed by SDS-PAGE followed by Western blotting with an anti-*myc* antibody. Full-length GBF1-*myc* was readily detected in the membrane and cytosolic fractions incubated at 37°C without added trypsin (Figure 5B, M* and C* lanes; band at ∼210 kDa). In addition, lower MW bands were detected in the M* fraction (bands at ∼170 kDa, ∼100 kDa, ∼66 kDa, ∼60 kDa and ∼56 kDa; marked with arrows in lane M*). The ani-*myc* recognizes cellular proteins other than the exogenously expressed GBF1-*myc* (see bands at ∼100 kDa and ∼56 kDa in the NT lane from untransfected cells and marked with red asterisks in all lanes), and those were excluded from subsequent analysis of digestion patterns. Interestingly, the bands at ∼66 kDa and ∼60 kDa detected in the membrane fraction (lane M*) were not detected in the cytosolic fraction (see lane C*), suggesting that membrane-associated GBF1-*myc* is more susceptible to proteolysis by endogenous proteases.

Addition of trypsin to the cytosolic fraction had minimal effect on GBF1-*myc,* and full-length GBF1-*myc* was detected after 15 and 30 min incubation (Figure 5B, C lanes). In contrast, addition of trypsin to the membrane fraction caused the digestion of GBF1-*myc* into three small fragments (Figure 5B, M lanes; bands at ∼66 kDa, ∼60 kDa and ∼54 kDa). These results suggest that the C-terminal *myc* within the cytosolic GBF1 is resistant to proteolysis and that upon binding to membranes, GBF1-*myc* assumes a more “open” conformation that exposes previously buried trypsin cleavage sites. The molecular sizes of the three *myc*-containing fragments in the membrane fraction suggest that the membrane-bound GBF1-*myc* is cleaved once within the C-terminus of the HDS2 domain at approximately amino acid 1259, and at two sites within the HDS2-HDS3 linker at approximately amino acids 1314 and 1369 (Figure 5D). GBF1 amino acid sequence contains putative trypsin cleavage site motifs (a K or R without adjacent C-terminal P) close to the detected cleavage sites. For example, K1252 is within 7 amino acids of the predicted cleavage at amino acid 1259; R1314 is at exactly the predicted cleavage at amino acid 1314; and K1351 and K1387 are within 18 amino acids of the predicted cleavage site at amino acid 1369 (Table 1).

**TABLE 1:** Characteristics of the cleavage sites unmasked upon GBF1 binding to Golgi membrane. Shown are the calculated cleavage sites that correspond to cleavages observed in Figure 5 (sites in green are calculated from the cleavage of GFP-GBF1 probed with anti-GBF1 and sites in purple are calculated from the cleavage of GBF1-Myc probed with anti-myc). Trypsin cleavage motifs (K or R without adjacent P) within the sequence of GBF1 present within 15 amino acids of the predicted cleavage site are listed, with the sites closest to the calculated cleavage site in red. Position of each predicted cleavage site within GBF1 is indicated, with those localizing within structured domains in blue. All predicted cleavage site are either surface exposed or within unstructured linker regions. The possible residues that may mask the predicted cleavage sites within structured domains (DCB, HUS, Sec7d, and HDS2) were determined based on AlphaFold predictions of nearest neighbors.

Calculated cleavage site	Adjacent trypsin cleavage site	Position within GBF1	Structural characteristics	Masked in cytosolic GBF1 by interactions with
178	R166; R172; R178; R196	DCB; immediately N-terminal to α-helix 6	surface exposed	C168 in α-helix 6; amino acids 171-176 in a loop between α-helix 6 and 7 of DCB
342	K344	linker between DCB and HUS	unstructured	
469	R459; R469; R477; R487	HUS; loop between α-helix 3 and 4	surface exposed	likely protected by dimerization
605	K595; K604; K605; R620	linker between HUS and Sec7d	unstructured	
642	R627; K633; R651; K658	linker between HUS and Sec7d	unstructured	
687	K674; K675; R677; R682; R691; K697; K699; K700; K701	linker between HUS and Sec7d	unstructured	
878	K859; K865; K880; R895	Sec7d; end of α-helix 10	surface exposed	Q837 in α-helix 8 and L851 in α-helix 9 of Sec7d
1259	K1251	HDS2; middle of α-helix 5	surface exposed	I1208 in α-helix 3 of HDS2
1314	R1314; R1331	linker between HDS2 and HDS3	unstructured	
1369	K1351; K1387	linker between HDS2 and HDS3	unstructured	

Additional insight into GBF1 conformational changes upon membrane binding was obtained by comparing cleavage patterns of cytosolic and membrane-bound GFP-GBF1 using an antibody directed against an internal GBF1 epitope contained within amino acids 1266-1379 (the exact position of the epitope is not known). Cells expressing GFP-GBF1 were fractionated into cytosol and total membranes, and each fraction was either incubated at 37°C for 30 min or supplemented with trypsin and incubated at 4°C for indicated times. The samples were then analyzed by SDS-PAGE followed by Western blotting with an anti-GBF1/1266-1379 antibody. As shown in Figure 5C, full-length GFP-GBF1 is readily detected in the membrane and cytosolic fractions incubated at 37°C without added trypsin (Figure 5C, M* and C* lanes). An additional band at ∼73 kDa is also detected in the M* and C* fractions, most likely representing the activity of an endogenous protease.

Addition of trypsin to the cytosolic fraction had minimal effect on GFP-GBF1, and full-length GFP-GBF1 was detected after 15 and 30 min incubation (Figure 5C, C lanes). This indicates that the N-terminal GFP within the cytosolic GBF1 is resistant to proteolysis and confirms the data shown in Figure 5A. In contrast, addition of trypsin to the membrane fraction resulted in the disappearance of the full-length GFP-GBF1 and the appearance of multiple smaller fragments (Figure 5C, M lanes; bands indicated by green arrows). This indicates that the membrane-bound GFP-GBF1 assumes a more “open” conformation that exposes previously buried trypsin cleavage sites and confirms the data shown in Figure 5A.

We estimated the MWs and the predicted number of amino acids within each fragment detected by anti-GBF1/1267-1379 (Figure 5C), based upon predicted trypsin sites in the primary sequence of GBF1. We considered that trypsin rapidly removes the GFP moiety from membrane-bound GFP-GBF1 (as shown in Figure 5A, M lanes) and predict that it cleaves membrane-bound GBF1-*myc* at amino acid 1259, 1314 and 1369 (as shown in Figure 5B, M lanes and Figure 5D). Because each detected fragment must contain the epitope contained within amino acids 1267-1379 all fragments must have their C-terminus at either amino acid 1314 (this fragment would contain 47 amino acids that could be recognized by anti-GBF1/1267-1379) or amino acid 1369 (this fragment would contain 102 amino acids that could be recognized by anti-GBF1/1267-1379). We considered the cleavage at 1314 to be slightly more frequent than that at 1369 (based on the intensity of the respective bands in Figure 5B), and for our calculations, we assumed that the fragments detected with the anti-GBF1/1267-1379 are those containing amino acid 1314 as their C-terminus.

Based on the MW and the predicted number of amino acids within each fragment detected by anti-GBF1/1267-1379, we approximated the location of the 7 newly exposed N-terminal cleavage sites (for example: to generate the 1136 amino acid fragment containing amino acid 1314 as the C-terminus, we would expect cleavage to occur at amino acid 178, etc.; Figure 5E). Four of the unmasked cleavage sites are predicted to reside within linker regions between the domains, and 3 unmasked cleavage sites are within structured domains (the DCB, HUS and Sec7d domains) (Figure 5E). Analysis of the GBF1 amino acid sequence for possible tryptic cleavage sites capable of generating each fragment finds a compatible lysine or an arginine within 10 amino acids of each predicted cleavage site (Table 1). All the K or R residues likely to represent the cleavage sites are predicted to be surface exposed, indicating that they must be masked within the cytosolic GBF1 by interacting with other residues within GBF1 (either intra- or inter-monomer), and that such interactions are broken during GBF1 binding to membranes.

### GBF1 is an antiparallel extended dimer with protruding HDS1-3 domains

**FIGURE 6:**
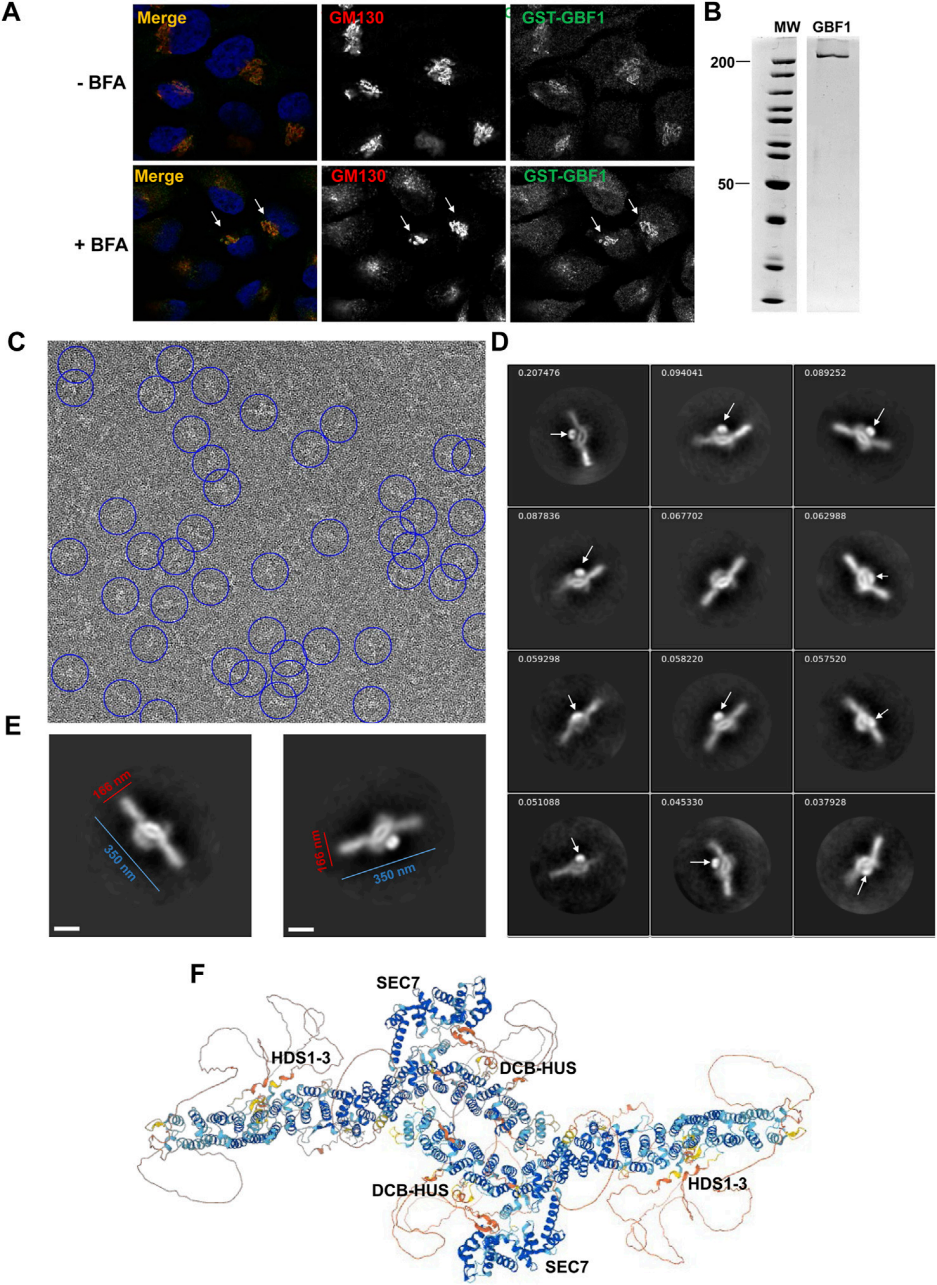
GBF1 is an anti-parallel dimer **(A)** HeLa cells were transfected with a BFA-resistant GST-tagged GBF1 and after 16 h media were replaced with fresh media without BFA (- BFA) or containing 0.5 μg/mL BFA (+BFA), cells were incubated for 30 min and then processed for IF to detect the GST tag and the GM130 Golgi marker. GST-GBF1 targets to the Golgi (-BFA panels) and is functional in maintaining compact Golgi architecture when the endogenous GBF1 is inactivated by BFA (+BFA panels, arrows). **(B)** An aliquot of GST-GBF1 purified from HEK cells was analyzed by SDS-PAGE and Coomassie blue staining. Two lanes of the same gel are shown. **(C)** A representative micrograph of negatively stained purified GST-GBF1 (lowpass filtered to 20 Å with contrast scaled to ± 3σ for clarity). A subset of GST-GBF1 particles included in the final 2D classes are circled in blue (corresponding to autopicking figure-of-merit ≥4). **(D)** A collage of representative GST-GBF1 2D classes labeled with the proportion of all 81,522 particles contributing to each class. The tightly folded GST moieties (arrows) mark the closely apposed positions of the N-termini of each GBF1 dimer. Bar represents 100 nm. **(E)** Dimensions of a GBF1 dimers. **(F)** AlphaFold predicted structure of GBF1 monomers assembled into a dimer based on the observed EM images.

The requirement for an intact HDS3 for GBF1 association with the Golgi, as documented above, prompted us to explore the position of the HDS3 domain within a full-length cytosolic GBF1 to inform possible engagement of HDS3 on the membrane. We generated an N-terminally GST-tagged human GBF1 (containing the Y828A mutation that confers BFA resistance to GBF1) and to ensure that the GST tag does not interfere with GBF1 functionality, we assessed the ability of GST-GBF1 to target to the Golgi and support Golgi homeostasis when expressed in HeLa cells in which the endogenous GBF1 is inhibited by BFA. As shown in Figure 6A, GST-GBF1 localizes to the Golgi and maintains Golgi architecture in cells treated with BFA.

GST-GBF1 was expressed in mammalian HEK cells, and the GST tag allowed efficient affinity purification (Figure 6B). Purified GST-GBF1 was subjected to negative staining and EM imaging (Figure 6C, representative image). Initial autopicking with Topaz followed by template-based autopicking, yielded 497,625 putative GBF1 particles. Reiterative 2D classification produced final 2D class averages from 81,522 total particles (Figure 6D, representative images). The obtained structures could be well fitted with two GBF1 monomers predicted by AlphaFold (compare Figures 6E, F). Correlating our structure with the AlphaFold prediction defined GBF1 as an antiparallel dimer with an oval central core and two protruding arms with overall dimensions of ∼350 nm in the longest axis and ∼166 nm for the central core (Figure 6E). The GST tag (Figure 6D, arrows) provides a positional marker for the N-termini of the two GBF1 monomers within the dimer, and their close apposition further supports previous findings that dimerization is mediated by the DCB-HUS domains of each monomer, based on the documented molecular interactions between these domains (Ramaen et al., 2007; Bhatt et al., 2016). The extended arms are formed by the HDS1-3 domains, while the Sec7d domains extend from the central core.

We correlated the structural information on the cytosolic GBF1 with the positions of the trypsin cleavage sites unmasked within membrane-bound GBF1 to gain insight into the possible conformational shifts occurring within GBF1 upon membrane binding (Table 1). The cleavage site within the DCB domain (at approximately residue 178) of membrane-bound GBF1 is buried within the cytosolic GBF1 by the C168 residue within the same α-helix 6 and by a loop (amino acids 171-176) between α-helix 6 and 7, suggesting that these DCB α-helices undergo inter-domain conformational shifts during GBF1 membrane binding. The cleavage site within the HUS domain (at approximately residue 469) that becomes exposed in membrane-bound GBF1 is within an exposed loop in cytosolic GBF1, suggesting that it is most likely masked by dimerization (Table 1). Its unmasking upon membrane binding may reflect at least a partial “breathing” or dissociation of the GBF1 dimer into a transiently looser or monomeric state on the membrane.

**FIGURE 7:**
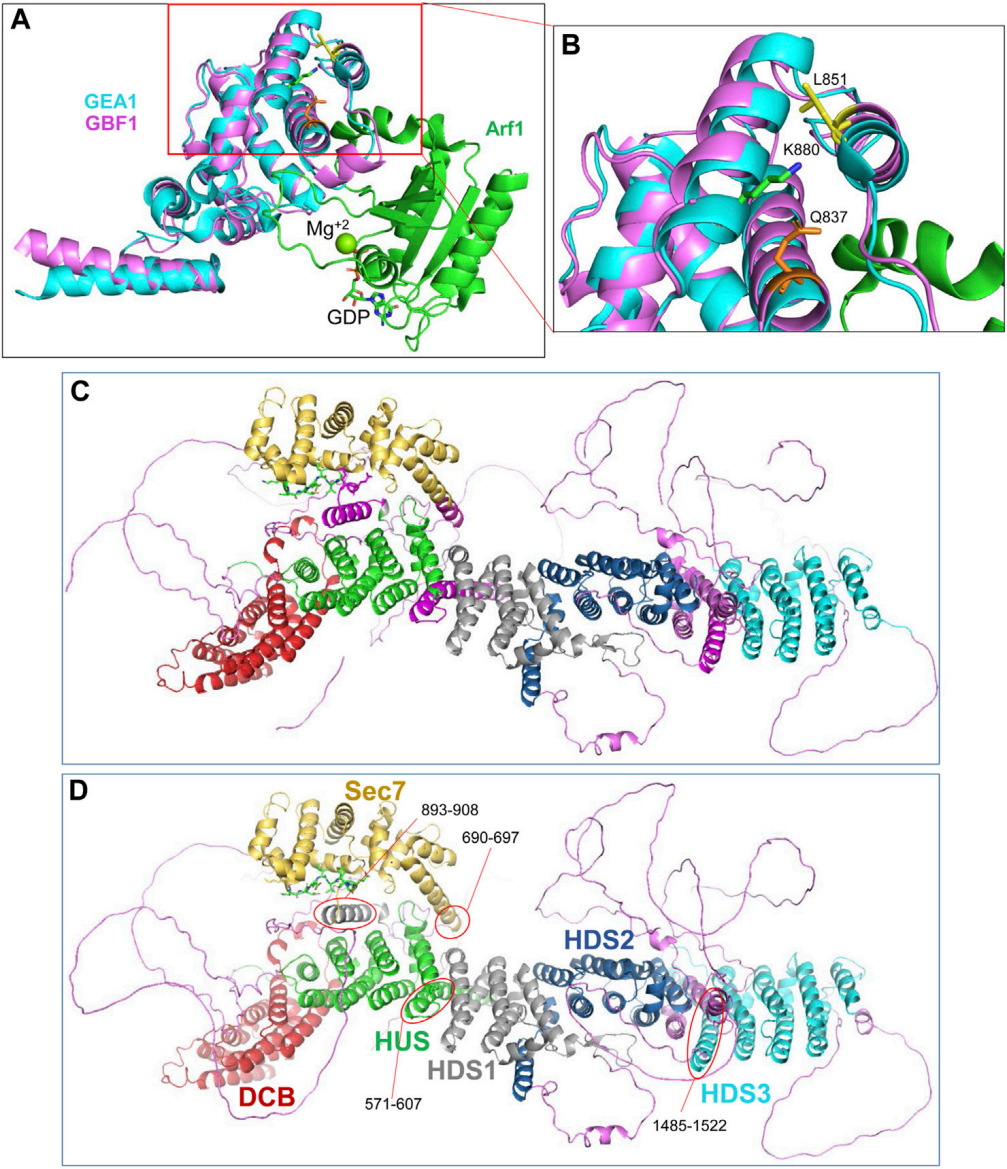
Domain organization within GBF1 **(A)** Overlay of the sequence of human GBF1 Sec7d on the crystal structure of the Sec7d from the yeast ortholog Gea1 bound to the substrate Arf1-GDP. **(B)** Enlarged area boxed in red in A shows the predicted cleavage site K880 in α-helix 10 unmasked during GBF1 membrane binding. It is masked in cytosolic GBF1 by Q837 in α-helix 8 and by L851 in α-helix 9 of the Sec7d, suggesting structural rearrangements of these helices relative to each other upon GBF1 binding to Golgi membrane. **(C)** AlphaFold predicted structure of a GBF1 monomer with domains color-coded as per domain assignment in Bui et al. (2009): DCB in red, HUS in green, Sec7d in gold, HDS1 in grey, HDS2 in blue and HDS3 in turquoise. Non-structured linker regions are magenta. Note that a number of α-helical regions shown in magenta were not assigned to a domain in that analysis. **(D)** As in C, but the previously non-assigned α-helices are marked by red ovals and color-coded based on their continuity with an assigned domain.

The cleavage site within the catalytic Sec7d (around residue 878; most likely at K880) is at the end of α-helix 10 (also called α-helix J), based on the threading of Sec7d of human GBF1 onto the solved structure of its Gea1p yeast ortholog (Mossessova et al., 2003; Renault et al., 2003) (Figure 7A). Alpha-helix 10 immediately precedes the “loop after J” shown by our prior crystallography work on the Sec7d of BIG2 to contact the Arf substrate during catalysis (Lowery et al., 2011). Importantly, an intact “loop after J″ is essential for GBF1 function, as substitution of the EIVMPEE residues at 883-889 within this loop with alanines inhibits GBF1 cellular function (the mutant targets to the Golgi, but acts as a dominant negative to tubulate the Golgi, and does not sustain Golgi morphology when endogenous GBF1 is inactivated with BFA) (Lowery et al., 2011). The K880 cleavage site is masked in cytosolic GBF1 by residue Q837 in α-helix 8 and residue L851 in α-helix 9 (Table 1; Figure 7B). The unmasking of K880 in α-helix 10 upon GBF1 membrane binding implies a conformational shift between this helix and α-helices 8 and 9 within the Sec7d.

The cleavage site within HDS2 (around residue 1259; most likely at K1252) is in the middle of the α-helix 5, and is protected by interactions with I1208 in α-helix 3 of HDS2 (Table 1). The remaining cleavage sites (at approximately 342, 605, 642, 687, 1314, and 1369) are within linker regions that are disordered within the AlphaFold-predicted structure and are also uncharacterized within our negatively stained GBF1 (Table 1). Yet, despite the lack of obvious structural content, these surface-exposed cleavage sites are efficiently masked in the cytosolic GBF1 dimer as to be trypsin inaccessible.

The correlation between the obtained EM structure and the AlphaFold prediction identified three α-helical regions that have not been previously recognized and/or assigned to a specific GBF1 domain (compare Figures 7C, D). Specifically, the helices marked in magenta in Figure 7C and circled in red in Figure 7D have not been previously assigned. Based on the correlation between our images and AlphaFold prediction, and circled in red in Figure 7D, an α-helix composed of residues 571-607 appears to be a part of the HUS domain, an α-helix composed of residues 893-908 appears to be a part of the HDS1 domain; and an α-helix composed of residues 1485-1522 appears to belong to the HDS3 domain Additionally, the α-helix 1 of GBF1 Sec7d is longer than previously reported (Bui et al., 2009) and includes amino acids 690–697 (Figure 7D, circled in red).

**FIGURE 8:**
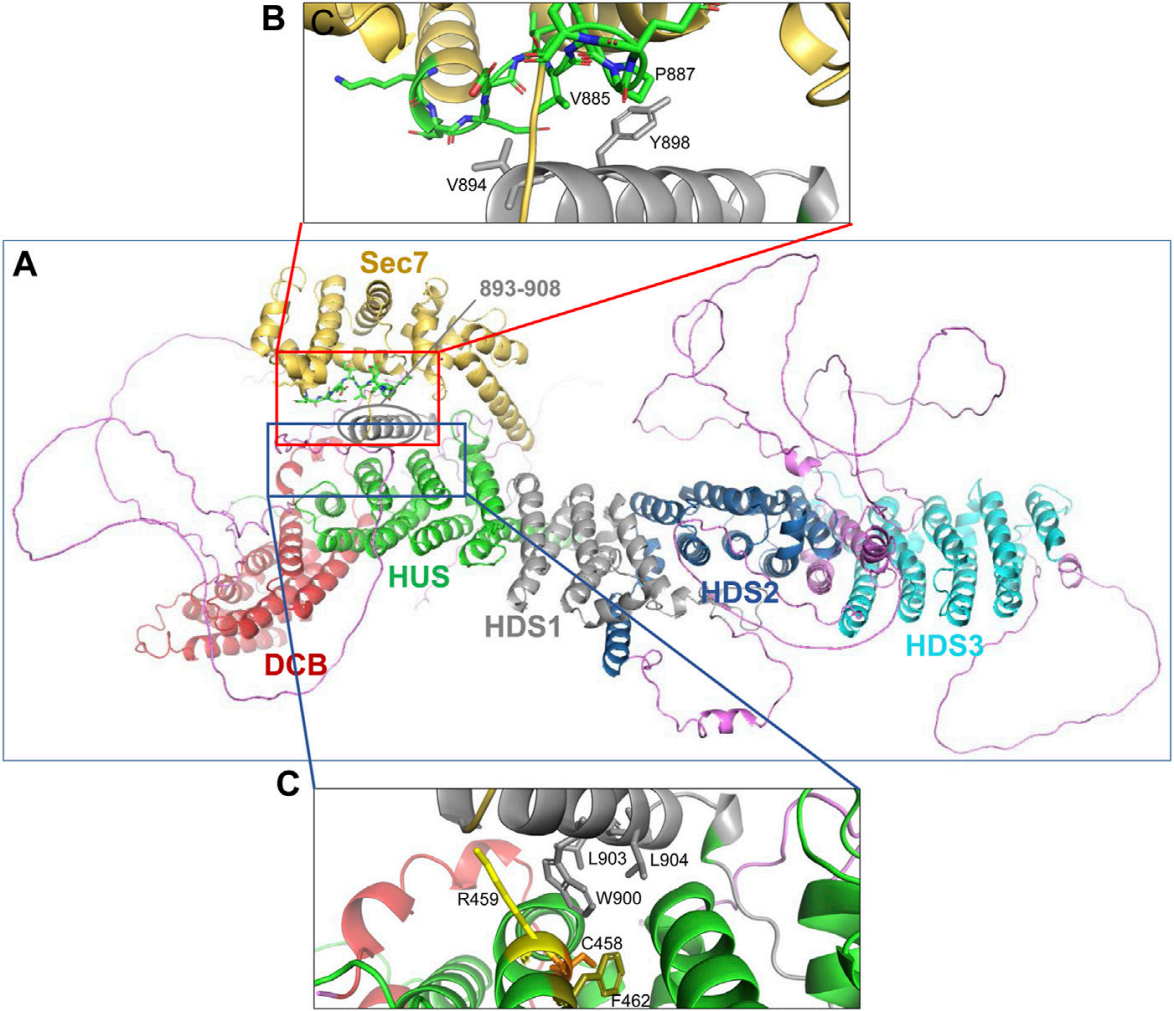
Domain interactions within GBF1 **(A)** AlphaFold predicted structure of a GBF1 monomer with color-coded domains: DCB in red, HUS in green, Sec7d in gold, HDS1 in grey, HDS2 in blue and HDS3 in turquoise. Non-structured linker regions are magenta. **(B)** Enlarged area boxed in red in A showing the close apposition of residues V894 and Y898 within an α-helix newly assigned to HDS1 to V885 and P887 within the critical EIVMPEE (883–889) motif of the loop after α-helix 10 of the Sec7d. **(C)** Enlarged area boxed in blue in A showing the close apposition of W900, L903, and L904 within an α-helix newly assigned to the HDS1 domain to residues C458, R459, and F462 within the α-helix 6 of the HUS domain.

The AlphaFold predicted α-helix composed of amino acids 893-908 that appears to be part of the HDS1 domain is of particular interest. This α-helix is spatially separated from the rest of the HDS1, and instead makes close contacts with both the Sec7d (Figure 8A, red square) and the HUS domain (Figure 8A, blue square). The 893-908 α-helix contacts the Sec7d through V894 and Y898 making close contacts with the V885 and P887 residues within the “loop after helix J” (α-helix 10) of the Sec7d (Figure 8B). The V885 and P887 residues are part of the Sec7d EIVMPEE motif (residues 883-889) that is essential for binding the Arf substrate (Lowery et al., 2011). The 893-908 α-helix also makes contact with the HUS domain via the centrally located W900 which represents a center of interactions, as it makes contacts with R459, C458, and F462 in α-helix 3 of the HUS domain (Figure 8C). The W900 position is stabilized by being sandwiched on one side by L903 and L904 within its own helix.

## Discussion

Herein, we have explored the structural determinants within the human Arf GEF GBF1 required for its function in maintaining Golgi homeostasis and secretion. GBF1 is a large (∼204 kDa) protein with multiple domains that are highly conserved across distant orthologs (Cox et al., 2004; Bui et al., 2009). The roles of these domains in GBF1 localization, function, and protein interactions have been defined to a varying extent. The central Sec7d is responsible for the binding of the substrate Arf and catalyzing the activating GDP/GTP exchange. The Sec7d of GBF1 has not been crystalized, but its sequence can be fit into the crystal structure of the yeast Gea1 ortholog (Goldberg, 1998) to provide an atomic level understanding of GBF1-mediated Arf activation (Figure 7A). Yet, the Sec7d alone is incapable of supporting Golgi homeostasis and secretion in cells, implying that the other domains of GBF1 are essential to provide the targeting and the regulatory information for GBF1 function *in vivo*.

The N-terminal DCB and HUS domains mediate GBF1 dimer formation (Ramaen et al., 2007; Bhatt et al., 2016), but dimerization is not required for GBF1 function, and instead seems to regulate its stability (Bhatt et al., 2016). The very N-terminus of GBF1 is required for membrane association (and hence cellular function), as the removal of 37 N-terminal amino acids or alanine substitutions of 10 N-terminal amino acids prevents GBF1 targeting to the Golgi (Viktorova et al., 2019). The roles of the C-terminal HDS1-3 domains in GBF1 function remain poorly understood. A fragment encompassing the HDS1 and HDS2 domains of GBF1 was shown to bind plasma membrane containing the products of PI3Kγ activity during neutrophil chemotaxis (Mazaki et al., 2012), and we documented that an intact HDS1 domain is required for GBF1 to bind PIPs and associate with Golgi membranes (Meissner et al., 2017). An intact HDS2 also is essential for GBF1 membrane binding and function *in vivo* (Pocognoni et al., 2018), albeit the molecular mechanism of its participation in GBF1 activity remains to be determined. The role that the HDS3 domain plays in GBF1 function has not been previously characterized.

The HDS3 domain is composed of 7 α-helices, with α-helices 1 and 3 containing stretches of residues that are highly conserved in GBF1 orthologs from humans to yeast (Figure 1A). We introduced mutations in three of the most highly conserved regions to generate the W1542A, PLL1544AAA, and FPL1594AAA mutants as well as a truncated GBF1 at amino acid 1531 that removes the entire HDS3 domain. The functionality of the HDS3 mutants was assessed in a cellular “replacement assay” to document that the FPL, PLL and 1531t mutants are severely (∼90% inhibition) compromised in sustaining Golgi architecture and cargo secretion (the W mutant is fully functional) (Figure 2). These phenotypes appear to be caused by the inability of the mutants to target to the Golgi (Figure 3), and imply that the HDS3 domain is a novel determinant of GBF1 membrane recruitment. Interestingly, the FPL, PLL and 1531t mutants retain the ability to associate with cellular membranes, as evidenced by both, immunofluorescence localization and fractionation experiments. These data suggest that an intact HDS3 domains is required for the selective targeting to the Golgi. This finding contrasts with the reported ability of a much shorter GBF1 fragment (the N-terminal 1–560 amino acid residues) to localize to the Golgi when expressed in cells (Mansour et al., 1998), but our experiments with the GBF1 mutant truncated at 560 did not detect Golgi targeting (data not shown).

The critical importance of the HDS3 domain in GBF1 function was further documented within an organismal context. The *Drosophila* gene gartenzwerg (garz) encodes the ortholog of mammalian GBF1 and is essential for embryogenesis (Wang et al., 2012). Moreover, the *garz* ^EMS667^ mutation, which truncates Garz within the HDS3 domain causes heart defects due to loss of Pericardin deposition in blood vessels (Wang et al., 2012). We further investigated the role of an intact HDS3 domain in Garz functionality by analyzing tubulogenesis in the salivary glands and the trachea of the *garz* ^*EMS667*^ mutant *Drosophila* embryos. The development of both organs was strongly inhibited, and the *garz* ^*EMS667*^ mutants did not hatch into larvae and died as embryos due to an inability to clear matrix from their tracheal tubes to allow gas exchange (Figure 4). In further support of the critical role of the HDS3 domain in organismal development, genetic analysis of a set of GNOM (a GBF1 ortholog in *Arabidopsis thaliana*) alleles revealed that truncation of GNOM protein at position 1369 (in the middle of the HDS3 domain (Bui et al., 2009)) causes inhibition in lateral root initiation (Geldner et al., 2004; Miyazawa et al., 2009). Together, these findings indicate that the removal of the C-terminal region containing the HDS3 domain of GBF1, Garz or GNOM dramatically interferes with their functionality, and define the HDS3 domain as a critical determinant of GBF1 membrane association and subsequent cellular activities.

Because GBF1 binding to Golgi membranes represents a key regulatory node in GBF1 cellular functionality, we aimed to gain insight into this process by examining the conformational changes induced within GBF1 upon membrane binding. We document that the association of GBF1 with membranes induces significant structural shifts, as reflected by the unmasking of 10 previously buried trypsin cleavage sites (Figure 5; Table 1). Membrane-induced changes in GBF1 architecture suggest that: 1) the HDS1 domain and the HDS2 domain (residues 911-1259) form a single tightly folded structural unit that retains its overall architecture during membrane binding as they remain trypsin inaccessible despite being joined by a linker region that contains two surface exposed trypsin cleavage sites at K1672 and R1690; and 2) The HDS1-HDS2 unit undergoes positional shifts relative to the rest of GBF1 as shown by the newly exposed cleavage sites immediately upstream of the HDS1 and within the C-terminus of the HDS2 (Figure 5).

To understand the spatial relationships between the distinct domains within human GBF1, we used negative staining electron microscopy to probe the structure of human cytosolic GBF1 purified from mammalian cells (Figure 6). The observed dimeric structure is in strong agreement with the architecture of monomeric GBF1 predicted by AlphaFold and with the structure of the GBF1 ortholog Gea2p from the yeast *S. cerevisiae* obtained by cryo-EM (Muccini et al., 2022). We used the structural information to correlate the domain organization within the cytosolic GBF1 with the unmasking of cleavage sites upon GBF1 membrane binding. Six of the unmasked 10 sites are within linker regions between domains and there is no structural information as they appear disordered in the AlphaFold-generated structure (Figure 6). Yet, these linkers must be at least partially structured to effectively mask the trypsin cleavage sites within the cytosolic GBF1, and these intrinsically disordered regions may nevertheless pattern after they engage membrane lipids and/or proteins to expose the cleavage sites. Moreover, the high frequency of structural rearrangements within the linker regions may suggest that they may act as hinges that regulate the membrane engagement of the more compacted and rigid conserved domains.

The cleavage sites within the structured domains (DCB, HUS, Sec7d, and HDS2) of GBF1 that are unmasked during GBF1 binding to membrane are all predicted to be surface exposed in the cytosolic GBF1, suggesting that they are masked through interactions with cognate partners. The masking can involve residues within the same domain or residues within other domains (Table 1). Importantly, their unmasking upon GBF1 membrane binding suggests strong conformational shifts within both, single domains, as well as changes of domain interactions and orientations relative to each other.

The availability of structural information on the full-length GBF1 allowed the identification of 3 previously unassigned α-helices as belonging to the HUS (amino acids 571–607), HDS1 (amino acids 893-908 and HDS3 (amino acids 1485–1522) domains (Figure 7). Additionally, the α-helix 1 of GBF1 Sec7d is longer than previously recognized and includes amino acids 690-697.

Of special interest is the newly assigned HDS1 α-helix as it makes close contacts with both, the HUS and the Sec7d domains (Figure 8). We have shown previously that the HDS1 domain is required for GBF1 membrane association by facilitating its interaction with Golgi PIPs (Meissner et al., 2017). It is interesting to speculate that the binding of HDS1 to membrane PIPs may induce conformational changes that impact the structure of the HUS domain (as shown in Figure 5D, GBF1 binding to membrane unmasks numerous cleavage sites within and bracketing the HUS domain) and simultaneously impact the arrangement of the catalytic Sec7d (as shown in Figure 5E, GBF1 binding to membranes uncovers cleavage sites bracketing the Sec7d). Such structural changes within the HUS, HDS1, and Sec7d might induce the optimal orientation of the GBF1 dimer on the membrane to effectively engage its Arf substrate prior to catalyzing the GDP/GTP exchange.

Understanding the folding of the cytosolic GBF1 and insight into the conformational changes occurring upon membrane binding is the first step in understanding the arrangement of GBF1 domains while membrane-bound. Future cryo-EM studies aimed at defining such structure are needed to understand the molecular mechanisms that facilitate distinct steps of GBF1 function, from membrane association through catalysis to dissociation. Such atomic level understanding of GBF1 is needed to advance our understanding of the spatio-temporal parameters of Arf activation that supports membrane flow at the ER-Golgi interface.

## Data Availability

The original contributions presented in the study are included in the article/supplementary material, further inquiries can be directed to the corresponding authors.
